# Beyond Accuracy: A MultiDimensional Framework for Evaluating Medical Image Classification Through Win vs. Lose Model Comparisons

**DOI:** 10.1007/s10278-025-01754-6

**Published:** 2025-12-02

**Authors:** Haixia Liu

**Affiliations:** University of the West of England Bristol, Gloucestershire, UK

**Keywords:** Medical image classification, Model interpretability, Grad-CAM, DermaMNIST, Performance evaluation, Deep learning, ResNet, RevNet, Win-Lose comparison

## Abstract

High-performing deep learning models such as ResNet, originally optimized for large-scale natural image datasets, often fail to generalize when applied directly to medical imaging tasks. This study investigates the limitations of “off-the-shelf” models in the context of skin lesion classification using the DermaMNIST dataset. Through a systematic evaluation of 35 architectural configurations across varying image resolutions and depths, the analysis reveals that mid-depth architectures (3–4 layers) and intermediate resolutions (128×128) achieve the best balance between accuracy and generalization. The top-performing RevNet-layer3 model (accuracy = 0.766) significantly outperforms deeper ResNet baselines (*p* < 0.05), demonstrating that increased depth or resolution does not guarantee improved medical domain performance. Beyond accuracy metrics, this study introduces a cross-architectural interpretability framework that compares Grad-CAM heatmaps between “Win” and “Lose” models, supported by quantitative perceptual metrics such as fractal dimension, entropy, and symmetry. The results show that fractal dimension consistently discriminates between effective and ineffective attention patterns, offering a more objective measure of model interpretability. These findings challenge the assumption of a universal top-performing model and highlight the need for domain-specific architectural probing and interpretability-driven evaluation. The work contributes a novel methodology for understanding performance–interpretability trade-offs in medical image classification, promoting more transparent and reliable deployment of deep learning systems in healthcare.

## Introduction

Medical image classification is a critical component of modern healthcare, particularly in dermatology for tasks like skin cancer detection. However, many studies in this field suffer from limitations in experimental rigor, often conducting single-run experiments to draw conclusions. This approach can lead to overgeneralized claims, as single-run experiments are prone to variability and may not account for the full spectrum of data scenarios or model behaviors. For instance, research by Renard et al. [26], Block et al. [7], and Bosma et al. [9] has demonstrated that deep learning approaches for medical image segmentation exhibit high variability, raising significant concerns about reproducibility. A single experimental run can produce misleading results in medical imaging contexts, given the inherent complexity and variability present in medical data. In response to these concerns, some studies have begun improving the robustness of their findings by conducting multiple experimental runs. For example, Liu et al. [17, 18] perform their experiments up to 10 times to better capture the variability inherent in medical imaging tasks and enhance the reliability of their conclusions.

Despite these progress, there remains a significant gap in the field: even with repeated experiments, there are still valuable insights left unexplored, particularly in the context of model interpretability, batch-specific performance, and more granular analysis of error patterns. This research aims to bridge that gap by providing deeper insights into model behavior through dynamic, interactive dashboards for explainable AI (XAI), leveraging batch-specific visualizations, confusion matrices, and more comprehensive investigations that go beyond the typical multiple-run experiments.

**Table 1:** Comparative analysis of methodological contributions

Aspect	Prior work	This work
Interpretability	Isolated Grad-CAM or feature map analysis	Cross-architectural pattern comparison (e.g., ResNet vs. RevNet)
	Single-architecture activation patterns [21]	
Localization Caution	Saliency maps in medical imaging require scrutiny [3]	Win model failure mode investigation. Boundary vs. interior attention comparison
	Correct prediction explanations [28]	
Art/spatial metrics	Symmetry evaluation [14]	Entropy-based heatmap analysis
	Texture analysis [12]	Fractal dimensions and spatial metrics

Recent developments in explainable artificial intelligence (XAI) have incorporated attribution-based methods such as Gradient-weighted Class Activation Mapping (Grad-CAM) as valuable tools for visualizing model attention in medical imaging applications, with studies demonstrating meaningful correlations between activation maps and clinically relevant features [25]. However, significant methodological limitations persist, including susceptibility to highlighting irrelevant artifacts and spurious correlations [16]. While some investigations have examined misclassified samples [8], these studies primarily focused on input-space perturbations rather than systematic activation pattern analysis.

Parallel research in fractal analysis has demonstrated that natural vision systems and effective neural networks exhibit similar geometric properties [31], while investigations into architectural constraints have explored enforcing symmetry principles on convolutional neural network filters inspired by biological vision and image processing paradigms. These approaches have shown that symmetry constraints significantly reduce free parameters while maintaining comparable image classification performance [10]. However, this research focused on assessing the impact of symmetrical filters in network architecture rather than on sample-level or feature map analysis. Contemporary work by Zhou et al. [35] has advanced multi-class vessel segmentation by addressing classification errors at vessel intersections through a dual-branch architecture that employs a binary segmentation branch to augment the primary multi-class network. This fusion methodology enhanced segmentation accuracy and established new performance benchmarks on clinical datasets, though it did not incorporate perceptual quality metrics.

These diverse approaches have remained largely disconnected from attribution-based interpretability methods, representing a significant gap in the comprehensive understanding of model behavior. This study addresses this limitation by developing a multidimensional analytical framework that systematically integrates attribution-based interpretability methods with misclassified sample investigation through comparative analysis of high-performing versus underperforming models, supplemented by traditional evaluation metrics. This comprehensive approach provides domain experts with granular insights into model decision-making processes across multiple analytical dimensions. Table 1 summarizes the key methodological contributions of this work compared to existing approaches across multiple analytical dimensions.

## Research Questions

This study investigates the following core aspects of skin cancer classification models:

**Fig. 1:**
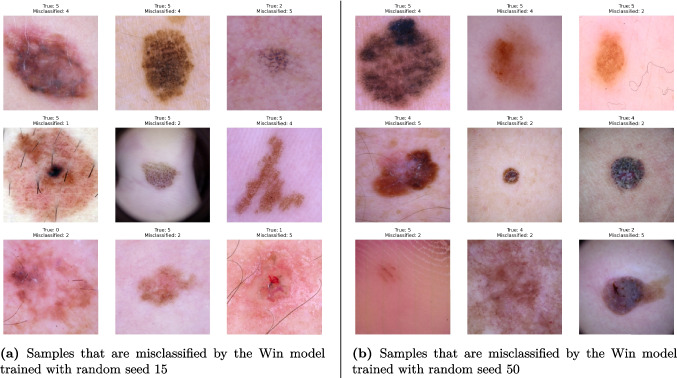
Sample images that are misclassified by the Win model

### Research Question 1: Model Performance Landscape

How do architectural variations, such as input resolution, network depth, and filter topology, in ResNet and RevNet architectures affect classification accuracy? ResNet is selected as a baseline due to its widespread adoption in computer vision applications. In contrast, RevNet, although less commonly used, has recently gained attention as a competitive alternative for medical image classification. Liu et al. [17] demonstrated that RevNet outperformed ResNet on the DermaMNIST dataset when filter placement was systematically modified. A subsequent study published by ACM (2024) [18] further showed that ResNet using Block2 at an input resolution of 64×64 achieved higher accuracy than deeper ResNet configurations operating at 224×224 resolution. By comparing a well-established residual architecture with a recently validated reversible counterpart, this study enables a systematic investigation into how specific architectural design choices influence both classification performance and model interpretability.

- **RQ1.1: Architectural Configuration Effects** Do systematic modifications to the ResNet-18 baseline (224×224 input, 4-block configuration, filter doubling) yield statistically significant accuracy improvements (p<0.05)? Validation employs Algorithm 1.
- **RQ1.2: Statistical Underperformance** Are there architectures with statistically significant underperformance (p<0.05) relative to the top performer when evaluated using Algorithm 2?

### Research Question 2: Visual Interpretability

How do Grad-CAM visualizations differ between high- and low-performing models? Grad-CAM was chosen because it produces class-discriminative localization maps directly from the final convolutional layers, providing clear visual insights into which image regions contribute most to a model’s decision. Unlike other methods (e.g., Integrated Gradients [24], layer-wise relevance propagation (LRP) [4], or saliency maps [1]), Grad-CAM combines interpretability with relative simplicity and computational efficiency, making it well-suited for cross-architecture comparisons on multiple models.

#### Differences Between High- and Low-Performing Models

Using Grad-CAM, high-performing (“Win”) models consistently focus on more structured and semantically relevant regions of lesions, such as central or diagnostically meaningful areas. In contrast, low-performing (“Lose”) models often highlight non-discriminative peripheral regions or fragmented patterns, even when making correct predictions. These systematic differences were quantified using perceptual metrics (fractal dimension, entropy, symmetry), with fractal dimension emerging as the most robust indicator of structured attention patterns.

- **RQ2.1: Attention Patterns in Misclassifications** Do the models that made the correct classification exhibit more clinically relevant attention (e.g., lesion boundary focus) in misclassified cases?
- **RQ2.2: Quantifying Interpretability** Can Grad-CAM outputs be objectively assessed via perceptual metrics (entropy, art-inspired metrics, spatial metrics)?

### Research Question 3: Batch-Level Performance Analysis

- **RQ3.1: Comprehensive Metric Comparison** How do the Win (128×128 RevNet-layer3) and Lose (224×224 ResNet-layer4) models compare across batch-level metrics including AUC-ROC, confusion matrices, and classification reports (precision/recall/F1)?
- **RQ3.2: Overfitting Investigation** To what extent does the performance gap between training and validation metrics indicate overfitting, and how does this differ between the Win and Lose models?

## Methods

### Dataset and Data Preprocessing

The study utilizes the DermaMNIST dataset [33] (10,015 RGB images across 7 classes) at multiple resolutions: 28×28, 64×64, 128×128, and 224×224 versions. All images underwent channel-wise normalization (μ=0.5, σ=0.5) via PyTorch transforms ,^1^ following standard pixel scaling [29]. The data was split with stratified sampling (stratify=y) into 60% training (6009 images), 20% validation (2003 images), and 20% test sets (2003 images), preserving original class distributions throughout all resolution variants. Some selected samples are shown in Fig. 1:

To ensure unambiguous interpretation of results, formally defined the class mappings are shown in Table 2, which bridges:

- **Model-facing** numeric labels (0–6)
- **Literature-standard** abbreviations

The DermaMNIST dataset contains **7 clinically distinct classes** of dermatoscopic images. Table 2 provides the complete reference mapping used in the experiments:

**Table 2:** The 7-class DermaMNIST label specification

ID	Abbr.	Clinical definition
0	akiec	Actinic keratoses and intraepithelial carcinoma
1	bcc	Basal cell carcinoma (malignant)
2	bkl	Benign keratosis-like lesions
3	df	Dermatofibroma (benign)
4	mel	Melanoma (malignant)
5	nv	Melanocytic nevi (benign moles)
6	vasc	Vascular lesions (angiomas/angiokeratomas)

**Fig. 2:**
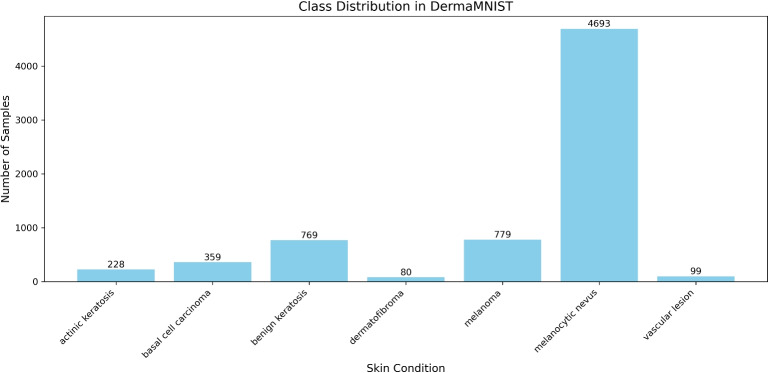
Class distribution histogram showing significant dataset imbalance

**Fig. 3:**
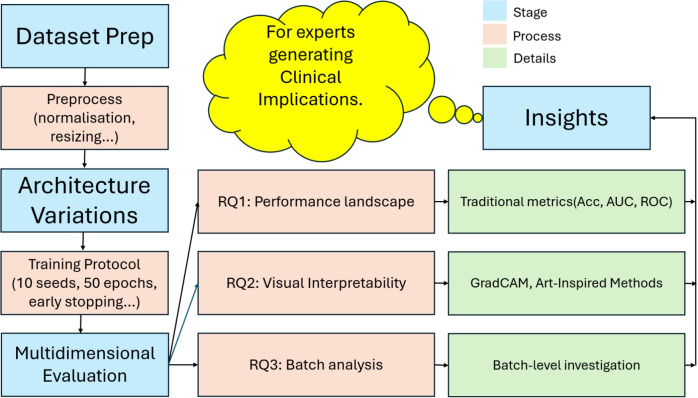
Overview of the proposed multidimensional evaluation framework for medical image classification

Figure 2 illustrates the class imbalance in the dataset, with the majority class (nv) compared to the minority classes (e.g., df and asc).

### A MultiDimensional Framework

A compact, multidimensional framework for evaluating medical image classification models was introduced, as illustrated in Fig. 3. The framework consists of three stages: dataset preparation, architectural variation, where novel architectures are constructed, and multidimensional evaluation. The final stage provides insights that are ready for expert inspection, focusing on comparisons between winning and losing models.

### Architecture Variations: FlexResNet

The proposed FlexResNet algorithm systematically explores the joint optimization space of input resolutions [18], network depth [18], and filter topologies [17] through three configurable dimensions: (1) *resolution scaling* across four levels (28×28, 64×64, 128×128, and 224×224 pixels), (2) *depth tuning* via four modular blocks (Block1-Block4) where each block adds 2 residual layers with skip connections, and (3) *filter topology* selection between standard ResNet and RevNet (reverse ResNet) configurations. This design yields 32 total architectural variants (4 resolutions × 4 depths × 2 topologies), enabling granular analysis of how resolution interacts with depth and filter design.

### Experimental Setup

All models were implemented in PyTorch, trained from scratch under identical conditions:

- **Training**: 50 epochs max, batch size 32, SGD (lr = 0.001, momentum = 0.9)
- **Validation**: Early stopping (patience = 10) monitoring cross-entropy loss
- **Reproducibility**: 10 runs per model with fixed random seeds (list_randseed=[1, 10, 15, 20, 25, 30, 35, 40, 45, 50])
- **Controls**: No pretrained weights or data augmentation

### Algorithms for Ranking Models

To systematically compare and interpret the performance of multiple models, a three-stage algorithmic framework was proposed. Algorithm 1 ranks models based on average accuracy and conducts statistical tests to identify significant performance differences. Algorithm 2 isolates models that perform significantly worse than the top-performing model. Algorithm 3 then identifies discriminative samples by comparing predictions across statistically distinct model pairs. This approach facilitates robust and interpretable model evaluation.

**Algorithm 1:**
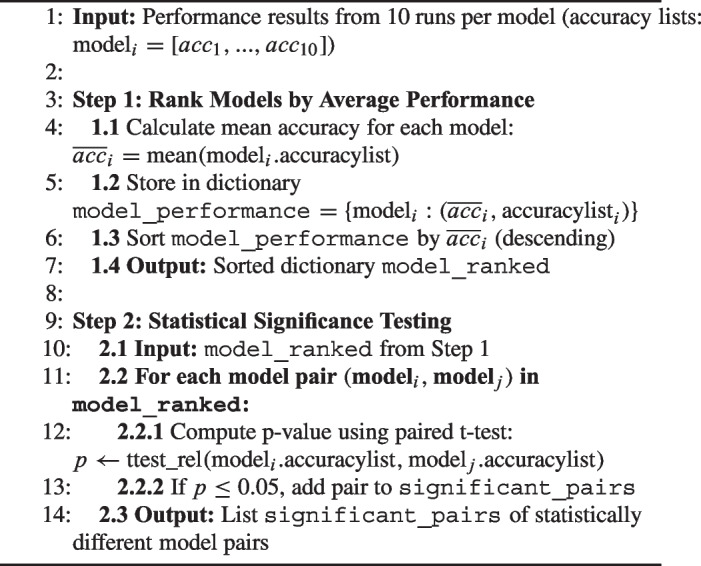
Ranked model performance comparison with statistical validation

**Algorithm 2:**
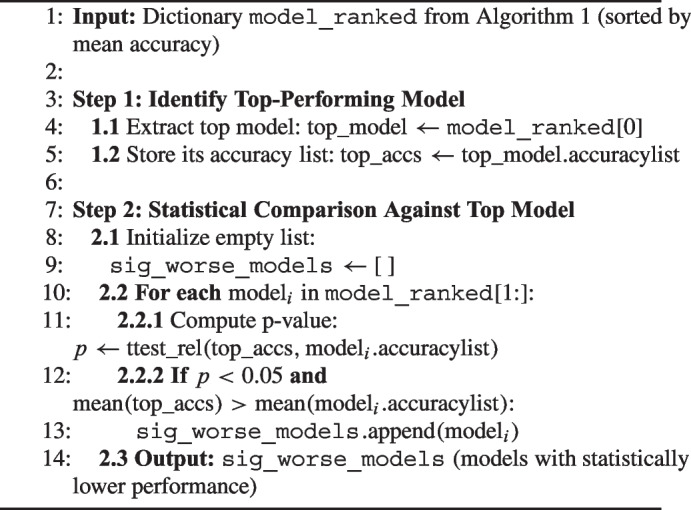
Identification of models significantly worse than top performer

**Algorithm 3:**
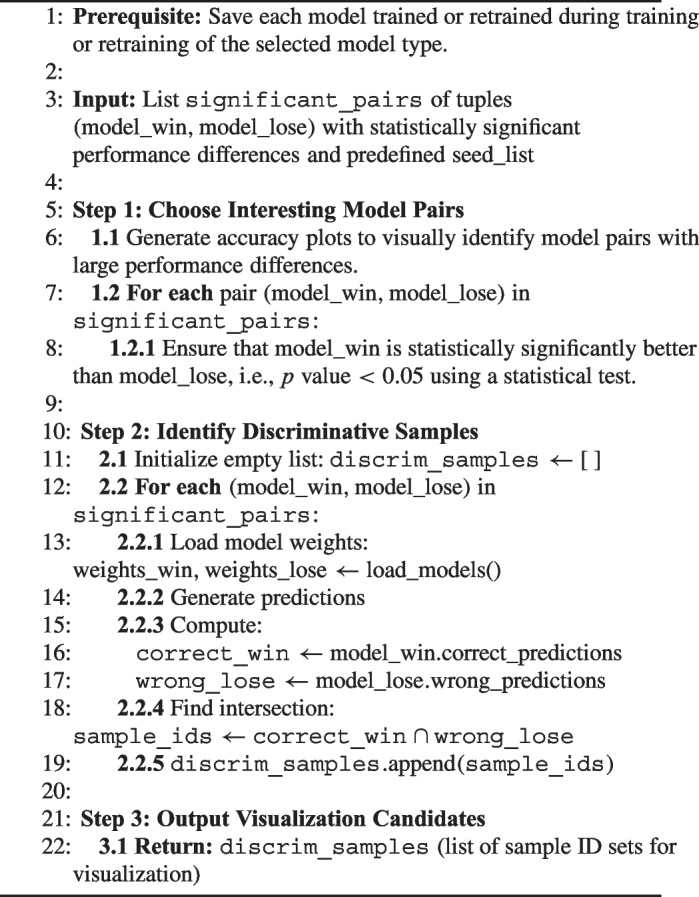
Significant sample selection for model comparison visualization

### Algorithms for Calculating Perceptual Metrics [23]

The implementation details of the algorithms used to address the research question (**RQ2.2**) are described below. All the following algorithms take the Grad-CAM heatmap as input and provide objective perceptual metrics for evaluating them.

#### Entropy

The entropy of the Grad-CAM activation map was computed by first calculating its normalized histogram over 256 bins spanning the range [0, 1]. To ensure numerical stability and avoid the logarithm of zero, a small constant was added to each histogram bin. The entropy was computed from the normalized histogram of the Grad-CAM activation map using the Shannon entropy formula implemented in the scipy.stats.entropy function from the SciPy library.^2^ A small constant was added to the histogram bins to avoid the logarithm of zero. This method captures the uncertainty in the distribution of activation values. Histogram-based entropy is a widely used metric in image processing and texture analysis [30].

#### Fractal Dimension

**Fig. 4:**
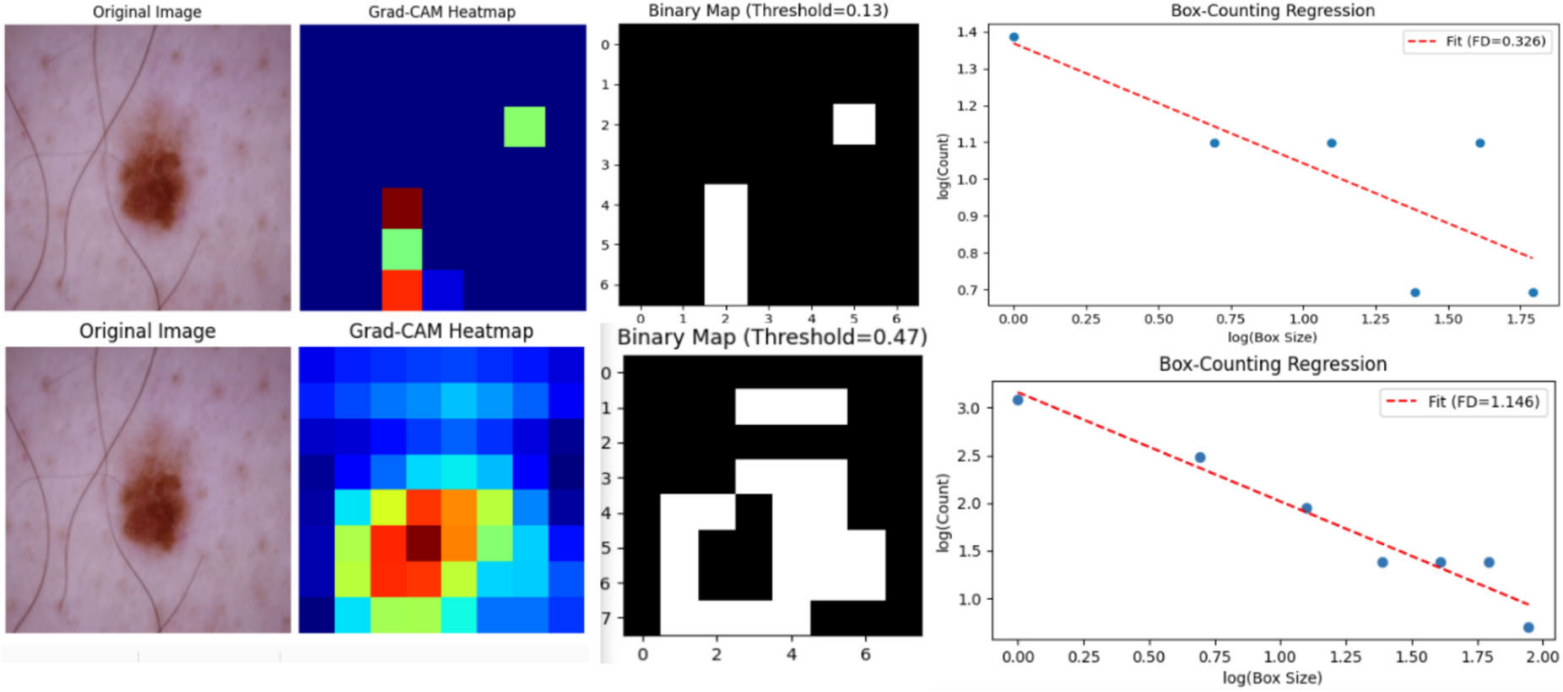
Composite output showing original image, Grad-CAM heatmap, binary thresholded map, and log-log regression for fractal dimension estimation

measures the spatial complexity of a Grad-CAM activation map using box-counting. After binarizing the map via Otsu thresholding, the algorithm counts how the number of active boxes scales across different box sizes. The negative slope of the log-log plot of box size versus count yields the fractal dimension, quantifying self-similarity and fine-grained structure in the activation. The algorithm presented in Algorithm 4 is adapted from the method proposed in [5]. This algorithm computes the fractal dimension of a Grad-CAM activation map using box-counting after thresholding. When plot = True in Algorithm 4, the corresponding visual outputs are generated, as illustrated in Fig. 4.

**Algorithm 4:**
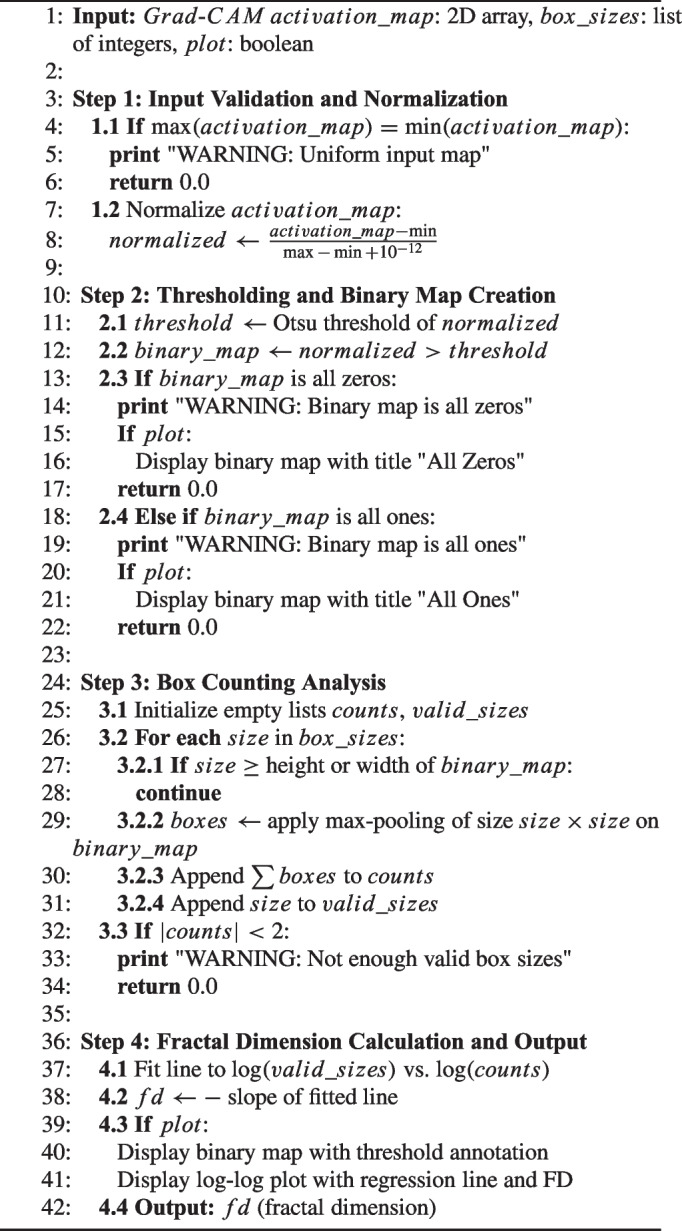
Calculate fractal dimension

#### Symmetry

quantifies how symmetric a Grad-CAM activation map is with respect to a specified axis (horizontal, vertical, or both) by computing the Pearson correlation coefficient between the normalized map and its flipped version [19].

**Algorithm 5:**
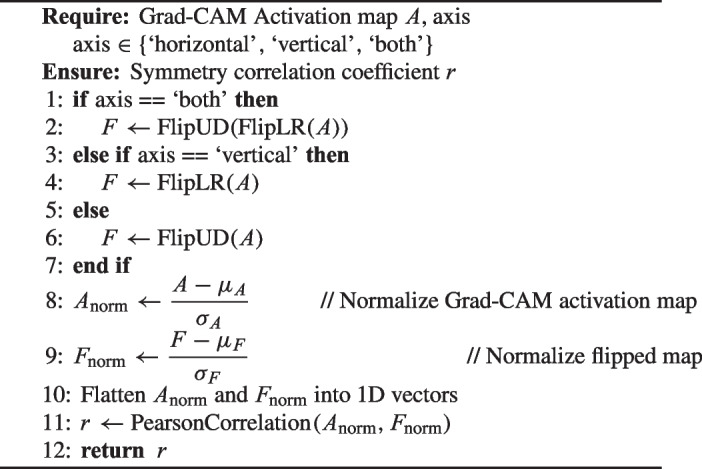
Symmetry correlation of Grad-CAM activation map [19]

#### Center of Mass Distance (COM Dist)

measures how far the center of mass of a Grad-CAM activation map is from the center of the image, using Euclidean distance [20].

**Algorithm 6:**
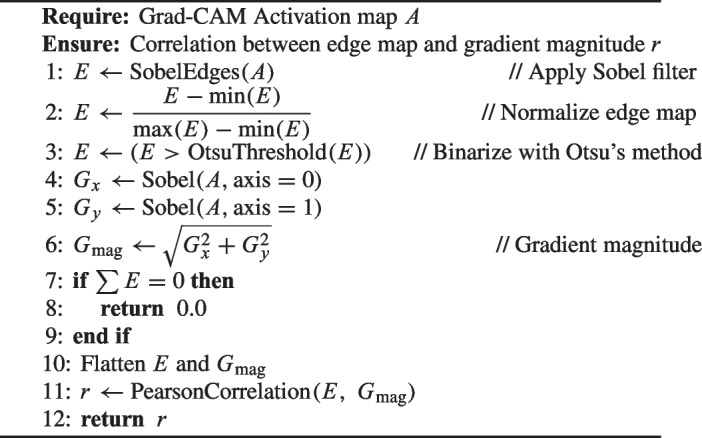
Center of mass distance (COM Dist) [20]

#### Boundary

The correlation between a binarized Sobel edge map and the gradient magnitude of the Grad-CAM activation map was calculated using Algorithm 7. Edges are extracted using the Sobel operator^3^ and binarized with Otsu’s thresholding method [22]. The gradient magnitude is computed from directional Sobel derivatives, and the Pearson correlation coefficient is used to quantify the alignment between the edge map and the gradient strength [27]. This specific formulation, correlating the edge map with gradient magnitude as a measure of structural alignment, is a novel contribution of this work.

**Table 3:** Model comparison groups and clinical significance

Comparison group	Clinical insight
WinCorrect vs LoseCorrect	Do high-performing models detect different visual biomarkers?
WinMis vs LoseMis	Are errors systematically different between strong and weak models?
WinCorrect vs LoseMis	Do correct calls in good models resemble errors in poor ones?
LoseCorrect vs WinMis	Can weak models succeed where strong models fail?

**Fig. 5:**
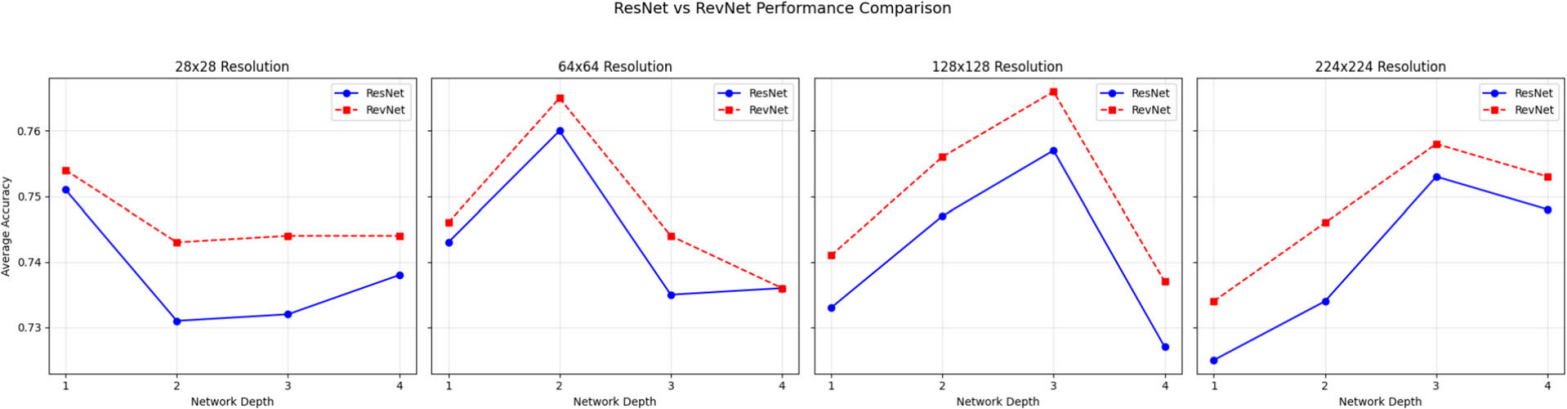
Performance (Average Accuracy of 10 Runs for DermaMNIST color images test set) comparing ResNet and RevNet architectures with different image sizes and neural network depths, using 10 different random seeds

**Algorithm 7:**
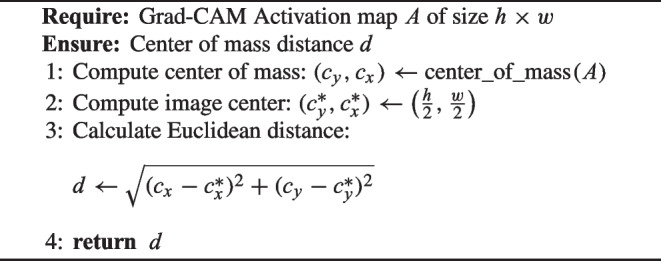
Boundary metrics: edge-aware gradient correlation

#### Edge density

Edge density is quantified by applying the Sobel filter to the image to detect edges, then computing the average edge strength across all pixels.

### Statistical Analysis and Multiple Hypothesis Correction

For each metric, the distribution between two groups was compared using three tests: Welch’s *t*-test (parametric), Mann–Whitney *U* test, and the Kolmogorov–Smirnov test. The Mann–Whitney *U* test was used by default to compute the feature-wise *p*-value due to its non-parametric nature. If both groups had n>30 samples, the Welch’s *t*-test *p*-value was used instead, assuming the central limit theorem applies. Multiple hypothesis testing^4^ was addressed using the Benjamini–Hochberg [6] false discovery rate (FDR) correction. Raw *p*-values were adjusted using Python’s multipletests function with α=0.05, and metrics with corrected *p*-values below this threshold were considered statistically significant. The full procedure is detailed in Algorithm 8.

**Algorithm 8:**
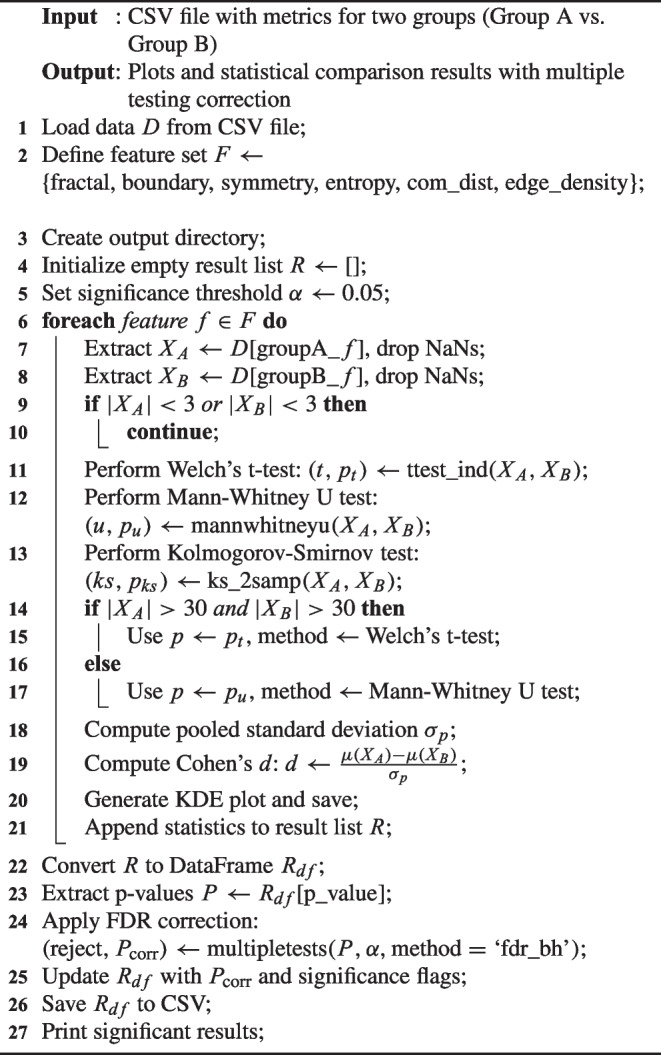
Statistical comparison of feature distributions between two groups

### Experimental Strategy

To investigate how model performance correlates with clinical relevance, a series of pairwise comparisons across models were conducted. These comparisons aimed to identify whether correct and incorrect predictions differ systematically between high- and low-performing models, and whether misclassifications in weaker models resemble successes in stronger ones. The experimental strategy is summarized in Table 3, outlining the clinical questions guiding each comparison group.

## Results

**Table 4:** Comparison of model performance between the winning model (128128rev_layer3) and all other models

Model	Architecture depth (layers)	Input resolution (pixels)	Filter topology	Accuracy	*p*-value
128128rev_layer3	3	128×128	rev	**0**.**766**	—
6464rev_layer2	2	64×64	rev	0.766	0.920
6464res_layer2	2	64×64	res	0.760	2.37e−01
224224rev_layer3	3	224×224	rev	0.758	9.34e−02
128128res_layer3	3	128×128	res	0.757	1.35e−01
128128rev_layer2	2	128×128	rev	0.756	8.32e−02
2828rev_layer1	1	28×28	rev	0.754	1.65e−02
224224rev_layer4	4	224×224	rev	0.753	2.17e−02
224224res_layer3	3	224×224	res	0.753	2.71e−02
2828res_layer1	1	28×28	res	0.751	9.40e−03
224224res_layer4	4	224×224	res	**0**.**748**	**1.80e**−**03**
128128res_layer2	2	128×128	res	0.747	2.04e−03
6464rev_layer1	1	64×64	rev	0.746	2.65e−03
224224rev_layer2	2	224×224	rev	0.746	1.11e−03
2828rev_layer3	3	28×28	rev	0.744	4.74e−04
2828rev_layer4	4	28×28	rev	0.744	2.13e−04
6464rev_layer3	3	64×64	rev	0.744	1.87e−03
2828rev_layer2	2	28×28	rev	0.743	1.76e−04
6464res_layer1	1	64×64	res	0.743	3.11e−03
128128rev_layer1	1	128×128	rev	0.741	7.71e−04
2828res_layer4	4	28×28	res	0.7381	4.89e−05
128128rev_layer4	4	128×128	rev	0.737	4.79e−05
6464rev_layer4	4	64×64	rev	0.736	4.75e−05
6464res_layer4	4	64×64	res	0.736	2.19e−04
6464res_layer3	3	64×64	res	0.735	6.55e−05
224224res_layer2	2	224×224	res	0.734	5.29e−06
224224rev_layer1	1	224×224	rev	0.734	1.61e−05
128128res_layer1	1	128×128	res	0.733	8.88e−06
2828res_layer3	3	28×28	res	0.732	4.33e−06
2828res_layer2	2	28×28	res	0.731	3.46e−06
128128res_layer4	4	128×128	res	0.727	5.77e−07
224224res_layer1	1	224×224	res	0.725	5.47e−07

The table shows average accuracy over 10 runs and statistical significance (*p*-values) for comparisons against the winning model. The best-performing (winning) model and the selected losing model (224224res_layer4) are highlighted in bold

### Key Findings for Research Question 1

### Model Performance Landscape

The experimental results shown in Fig. 5 and Table  4 reveal several statistically significant trends (p<0.05) across architectural variations:

- **Optimal Configuration**:
  - *Resolution*: 128×128 inputs outperformed both lower (28×28) and higher (224×224) resolutions
  - *Depth*: 3–4 layer architectures achieved peak accuracy (0.766 for RevNet-layer3)
  - *Topology*: RevNet showed a marginal advantage over ResNet in top-performing models
- **Statistical Significance**:
  - The top-performing configuration (128×128 RevNet-layer3, accuracy = 0.766) significantly outperformed 74% of all tested variants (26/35, p<0.05, Algorithm  1).
  - Top1 configuration 128×128 RevNet-layer3 is significantly better than the traditional 224×224 ResNet-layer4 with p<1.80e-3.

#### Performance Variance

 

- Intermediate resolutions (64×64, 128×128) showed ∼1.3% accuracy gain over extremes (28×28, 224×224):
  1 $$\begin{aligned} \text {Gain}&= \frac{\text {Best}_{\text {intermediate}} - \text {Mean}_{\text {extremes}}}{\text {Mean}_{\text {extremes}}} \times 100\% \nonumber \\&= \frac{0.766 - 0.756}{0.756} \times 100\% = 1.32\% \end{aligned}$$
- Depth increased performance up to layer 2–3, with diminishing returns beyond:
  2 $$\begin{aligned} \text {Layer1}_{\text {best}}&= 0.754\end{aligned}$$
  3 $$\begin{aligned} \text {Layer2}_{\text {best}}&= 0.766 \quad (+1.59\%) \end{aligned}$$
  4 $$\begin{aligned} \text {Layer3}_{\text {best}}&= 0.766 \quad (+0.00\%) \end{aligned}$$
  5 $$\begin{aligned} \text {Layer4}_{\text {best}}&= 0.753 \quad (-1.70\%) \end{aligned}$$

**Fig. 6:**
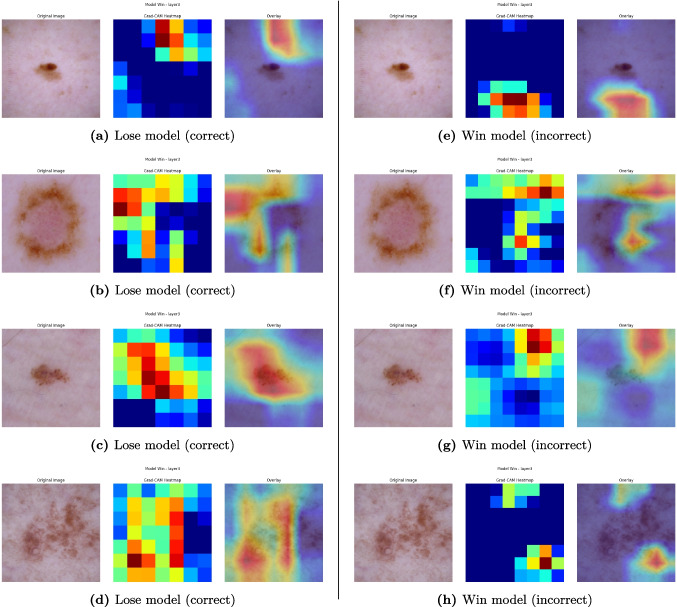
Comparison of model visualizations: the left column shows FeatureMaps and Grad-CAM visualizations generated by the Lose model, which correctly identified the sample labels. The right column shows the same samples visualized by the Win model, which misclassified them

### Direct Answers to Research Sub-Questions

1. **RQ1.1**: Architectural modifications significantly impact accuracy. The optimal configuration combines:
  Mid-range resolution (128×128)
  Moderate depth (3–4 layers)
  RevNet topology
2. **RQ1.2**: Statistical analysis confirms:
  **Win model** (128×128 RevNet-layer3) significantly outperforms the **Lose model** (224×224 ResNet-layer4) by +0.018 accuracy ($p = 1.80 \times 10^{-3}$)

**Fig. 7:**
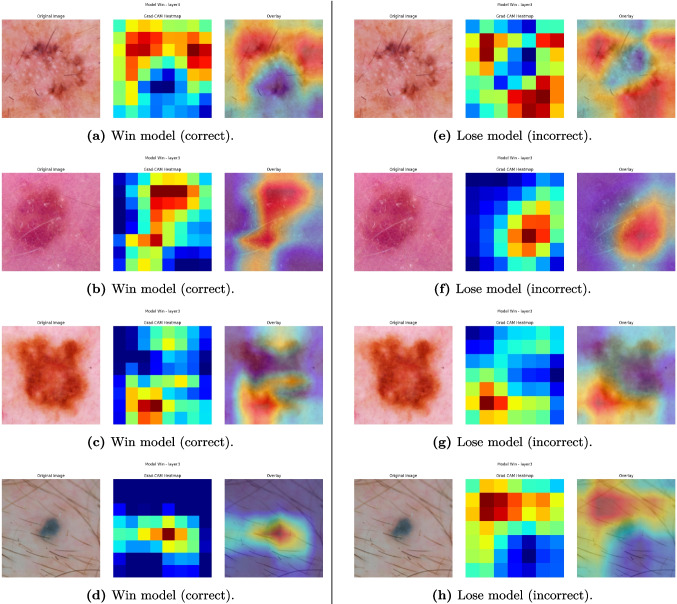
Comparison of model visualizations: the left column shows FeatureMaps (middle) and Grad-CAM (right) generated by the Win model which correctly identified the sample labels. The right column displays the same samples with their FeatureMaps and Grad-CAM visualizations derived by the Lose model which misclassified these samples. Grad-CAM heatmaps use a color scheme where warmer colors (red/yellow) indicate high model attention, and cooler colors (blue) indicate low attention

**Fig. 8:**
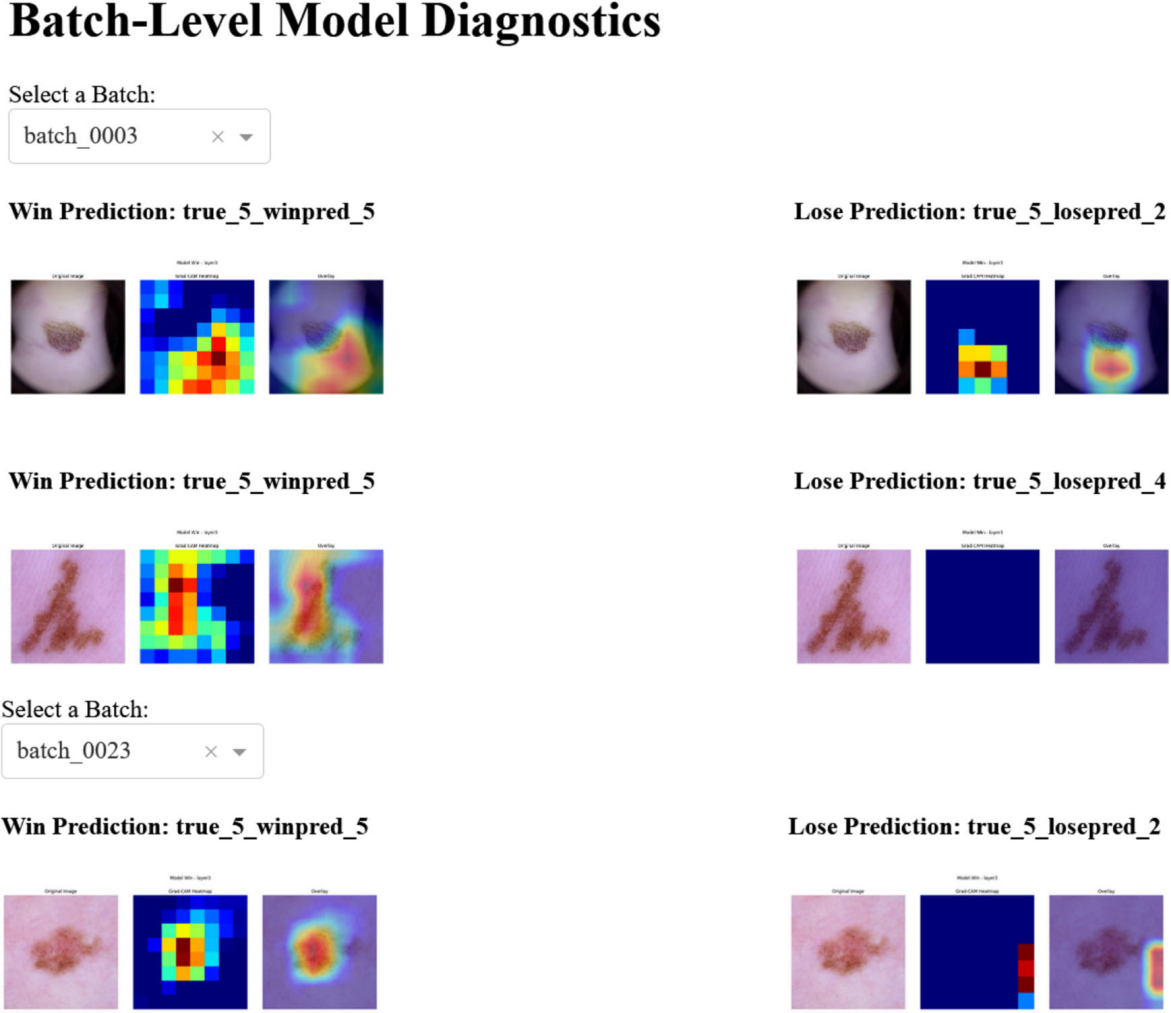
Interactive model comparison tool visualizing misclassified samples with feature maps and Grad-CAM

### Findings for RQ2.1: Attention Patterns in Misclassifications

In Grad-CAM heatmaps (i.e., the class-discriminative activation maps), the intensity of activation is visualized through a colormap, where red and yellow indicate strong positive influence on the model’s decision, and blue signifies regions with negligible contribution. Figure 6 presents three representative challenging cases that demonstrate distinct failure patterns between correct and incorrect classifications. In these examples, the Win model misclassified the lesions while the Lose model achieved correct predictions.

#### Top Row

The lesion has a relatively dark center and sharp contrast with the surrounding skin. Both models fail to accurately localize the lesion. However, the Lose model made the correct classification.

#### The Second Row

The lesion exhibits a diffuse, solar eclipse-like appearance with prominent outer boundaries and minimal central features. The Win and Lose models demonstrate irregular attention patterns, focusing on different boundary segments rather than systematic lesion coverage.

#### The Third Row

The lesion is small, compact, and roughly oval to irregular in shape. The Lose model (left) localizes the lesion but highlights extended regions, with attention concentrated along partial boundaries. In contrast, the Win model (right) misclassifies the lesion, disproportionately attending to non-discriminative peripheral regions.

#### The Fourth Row

The lesion exhibits a sprawling growth pattern, with multiple connected components and satellite areas. The Lose model (left) successfully captures several internal regions of the lesion. In contrast, the Win model (right) highlights only two separate peripheral areas that barely intersect with the true lesion boundaries, which likely explaining its misclassification.

Figures 7 shows a similar Win/Lose model comparison where the Win model correctly classified the lesions while the Lose model failed. Together, Figs. 6 and 7 suggest that

- Attention to non-pathological regions can occur in both accurate and erroneous predictions
- The spatial distribution of attention features alone may not fully explain model performance

An interactive dashboard (Fig. 8) was developed to enable granular comparison of decision-making patterns between the Win and Lose models by visualizing misclassified samples alongside their feature maps and Grad-CAM heatmaps. Figure 8 illustrates three representative challenging cases that highlight distinct failure patterns between correct and misclassified predictions. The top case presents a lesion with dark background edges, where both models focus on boundary regions but exhibit different attention distributions, the Win model shows more dispersed feature activation compared to the concentrated activation of the Lose model. The middle case features an irregularly-shaped lesion resembling a signature pattern, where the correct model successfully attends to target regions while the incorrect model fails to identify the lesion area. The bottom case displays an asymmetrical lesion with an irregular, poorly defined border where the Win model focuses on boundary regions with a slight inward offset from the lesion edges, whereas the misclassifying model largely misses the target with only minimal attention at one edge. Distinguishing between lesion classes requires more comprehensive information, including color, texture, and other contextual features. The degree of confusion between classes can vary significantly depending on these factors.

**Fig. 9:**
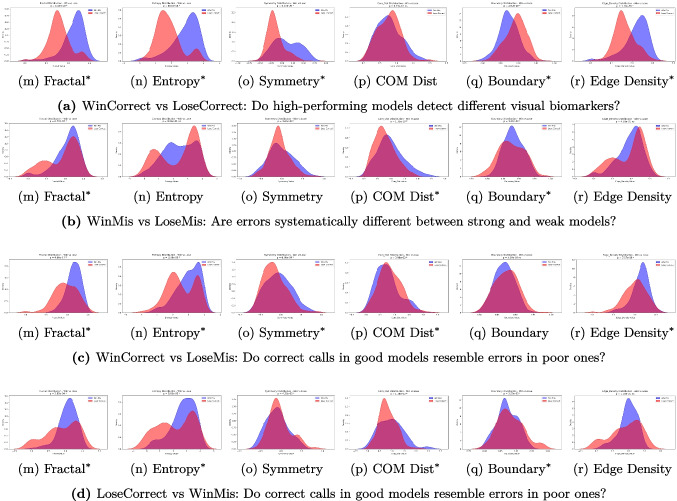
Metric comparisons across model groups. *Indicate statistical significance, based on the Algorithm 8

### Findings for RQ2.2: Quantifying Interpretability Through Perceptual Metrics

Grad-CAM heatmaps were objectively evaluated using perceptual metrics. The results are presented in Fig. 9.

- **Entropy (column n) emerges as a key discriminative metric:**
  - Shows statistical significance (∗) in **3/4 comparisons** (Fig. 9a, c, d)
- **Art-inspired metrics (columns m and o) reveal distinct patterns:**
  - **Fractal Dimension (m)** is universally significant (∗ in all comparisons)
    - Captures systematic differences in attention patterns across *all scenarios* (correct/erroneous predictions)
    - The most consistent metric in the analysis
  - **Symmetry (o)** shows selective significance (∗ in Fig. 9a and c)
- **Spatial metrics (columns p–r) offer niche insights:**
  - **COM Distance (p)**: Significant in **3/4 comparisons** (∗ in Fig. 9b, c, and c)
  - **Boundary (q)**: Significant in **3/4 comparisons** (∗ in Fig. 9a, b, and d)
  - **Edge Density (r)**: Significant in **2/4 comparisons** (∗ in Fig. 9a and c)

**Fig. 10:**
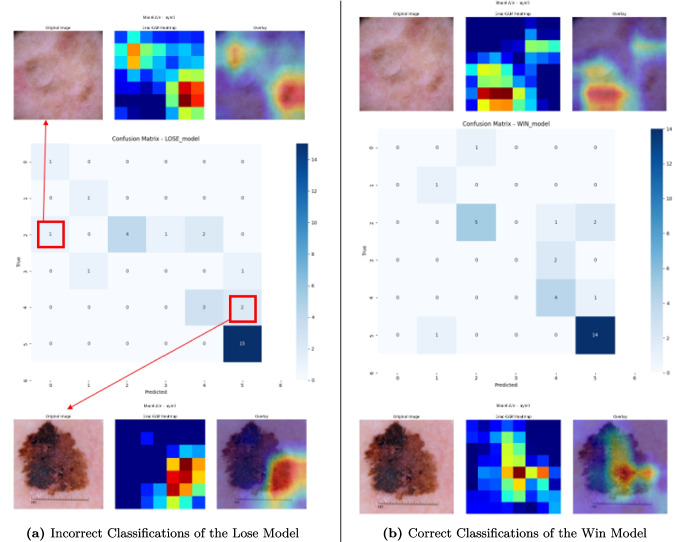
Comparative analysis of a randomly selected batch (seed = 50). **Right**: confusion matrices showing the Win model’s performance, with fewer misclassifications (e.g., true class 4 → predicted 5: Lose model’s 2 cases vs. Win model’s 1 case). However, Win model misclassified one sample as class 1, but the true class label is 5, which was correctly identified by Lose model. **Top and Bottom**: representative samples where the Win model correctly classifies lesions. The Grad-CAM comparison reveals distinct attention patterns—the Win model (bottom right) focuses on central lesion regions, while the Lose model (bottom left) concentrates activations in a peripheral quadrant of the lesion

## Closing Analysis for RQ2.2

- **WinCorrect vs LoseCorrect:** When both the high-performing and low-performing models make correct predictions, the Grad-CAM heatmaps reveal that the visual patterns they rely on are systematically different. This is evident across most metrics, including fractal, entropy, symmetry, boundary, and edge density. However, metric COM Dist shows no significant difference, suggesting that both models tend to focus on similarly located regions, even if the quality or nature of attention differs.
- **WinMis vs LoseMis:** When both models make incorrect predictions, the results are mixed: half the metrics indicate no significant difference, implying shared failure modes.
- **WinCorrect vs LoseMis:** When high-performing models make correct predictions and low-performing models make errors, their Grad-CAM heatmaps show clear differences across most metrics, especially fractal, entropy, symmetry, COM Dist, and edge density. This suggests that better models attend to more structured and meaningful regions. However, the boundary shows little distinction.
- **LoseCorrect vs WinMis:** When low-performing models make correct predictions and high-performing models make mistakes, the Grad-CAM heatmaps do not reveal substantial systematic differences in some patterns. Metrics such as fractal, entropy, COM Dist, and boundary show significant divergence. Symmetry and edge density, however, show minimal variation.
- **Metric Robustness:** Comparing across metrics, **Fractal Dimension (m)** emerges as the most robust indicator. It consistently shows significant differences in all scenarios (marked with $^*$), outperforming even **Entropy (n)** in consistency and sensitivity to model performance differences.

**Table 5:** Side-by-side classification report comparison

	Win model			Lose model			
Class	Prec	Rec	F1	Prec	Rec	F1	Support
akiec	0.00	0.00	0.00	0.50	1.00	0.67	1
bcc	0.50	1.00	0.67	0.50	1.00	0.67	1
bkl	0.83	0.62	0.71	1.00	0.50	0.67	8
df	0.00	0.00	0.00	0.00	0.00	0.00	2
mel	0.57	0.80	0.67	0.60	0.60	0.60	5
nv	0.82	0.93	0.88	0.83	1.00	0.91	15
Macro	0.45	0.56	0.49	0.57	0.68	0.58	32
Wtd.	0.70	0.75	0.71	0.77	0.75	0.73	32

*Note*: *Prec.* precision, *Rec.* recall. Both models evaluated on identical test batches

**Fig. 11:**
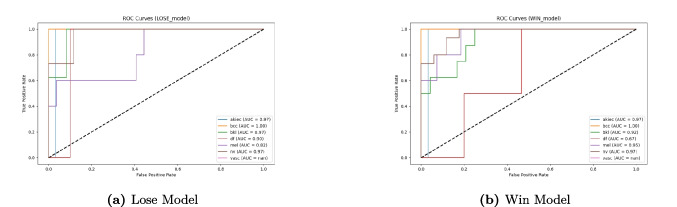
ROC curve comparison for selected batch (seed = 50). The Lose model shows stronger performance in DF (label = 4) class classification (AUC = 0.90 vs Win’s 0.67), while the Win model demonstrates superior detection of Mel (label = 2) class (AUC = 0.95 vs Lose’s 0.82). This divergence suggests complementary strengths: the Lose model better identifies DF cases, whereas the Win model excels at Mel detection

**Fig. 12:**
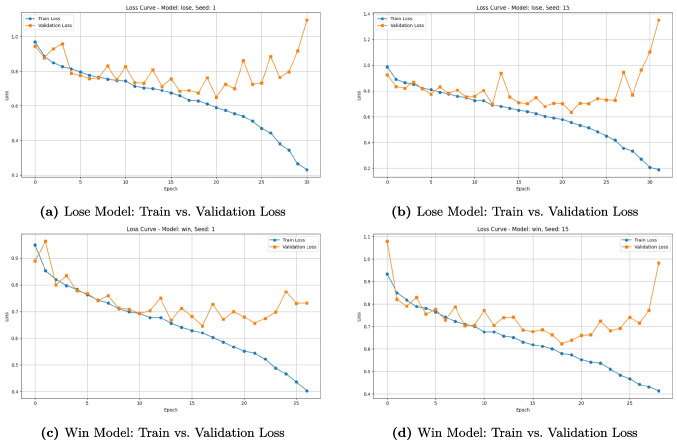
Training and validation loss curves for Win vs. Lose models. Clear overfitting observed in Lose model (top) with validation loss increasing from 0.66 to 1.17 after epoch 20, taking seed = 1 for example. Win model has an overfitting issue as well as evidenced on the bottom graphs but it is better than the Lose model

**Table 6:** Comparison of model performance across related studies

Model	Description	Size of train, val, test	Accuracy
ADKD2025 [34]	Adaptive Decoupled Knowledge Distillation	7007, 1003, 2005	0.82
DDPG-SARM2025 [2]	Deep deterministic policy gradient with self-adaptive reward mechanism	7007, 1003, 2005	0.79
VFL-CLIP2025 [32]	Combines vertical federated learning (VFL) with the CLIP (Contrastive Language–Image Pre-training)	7007, 1003, 2005	0.79
FuzzyEnsemble2025 [13]	Fuzzy ensemble of Xception, InceptionResNetV2, MobileNetV	4200, 600, 1200$^{a}$	0.78
Surrogate-Compress2024 [11]	Surrogatebased multiobjective compression method on three MedMNIST datasets	7007, 1003, 2005	0.76
FlexNet2025[This work]	Varying image resolution, neural network depth, and filter topology	6009, 2003, 2003	0.76
HNN-QC2025 [15]	Hybrid Neural Networks with Quantum Transformations	8000, 1000, 1000	0.73

Reported accuracy values correspond to the test set

$^{\text {a}}$The authors performed data balancing: the total sample size was 42,000, divided into training, validation, and testing sets with a ratio of 7:1:2. Each of the seven classes contained 6000 samples

### Findings for RQ3.1: Batch-Level Metric Comparison (Table 5, Figs. 10 and 11)

#### Batch-Level Error Analysis (Seed 50)

- **Win model advantage**: Higher recall for mel (0.80 vs 0.60)
- **Lose model advantage**: Perfect akiec recall (1.00 vs 0.00)
- **Shared limitation**: Zero df class detection
- **Trade-off**: Win better at mel, Lose better at rare classes

### Answer to RQ3.2: Win Model Is Not Perfect

Overfitting problems are shown in Fig. 12.

### Comparison with Existing State-of-the-Art Approaches

Comparison with existing state-of-the-art approaches is shown in Table 6. Our work still has limitations compared with the state-of-the-art methods from others, but the main focus of this paper is to provide insights that accuracy numbers alone cannot convey. It should be noted that some results may not be directly comparable because the training, validation, and test splits differ across studies.

## Conclusion, Discussion, and Future Work

### The Illusion of a Universal Top-Performing Model

This study demonstrates that even high-performing convolutional architectures optimized for natural image benchmarks (e.g., ResNet-18 trained on ImageNet) exhibit systematic failures when transferred to medical image analysis, particularly on diagnostically challenging dermatological samples. Despite achieving competitive accuracies, such models fail to generalize across variations in lesion morphology, texture, and illumination—exposing the illusion of a universal top-performing model.

### Summary of Key Findings in Context

Moderate-depth architectures (3–4 layers) and intermediate image resolutions (128×128) achieved statistically superior performance compared to deeper or shallower networks and extreme resolutions. The RevNet topology slightly outperformed ResNet, suggesting that architectural reversibility may enhance feature stability. These findings partially align with prior work on depth–resolution trade-offs and the rearrangement of filter placement [17, 18], but extend existing studies by systematically quantifying performance across 35 configurations with statistical validation.

Previous research has often focused on either benchmarking single architectures or generating post-hoc saliency maps. This study bridges these approaches through *cross-architectural interpretability analysis*, comparing Grad-CAM patterns bet-ween “Win” (high-performing) and “Lose” (low-performing) models on identical samples. This enables a deeper understanding of *where* and *why* models fail, addressing prior gaps that treated misclassifications as isolated events rather than structured phenomena.

### Novel Contributions

- **Cross-architectural interpretability:** The first systematic comparison of Grad-CAM heatmaps across architectures, revealing that high-performing models attend to more structured and semantically meaningful regions.
- **Quantitative perceptual analysis:** Introduction of art-inspired metrics (fractal dimension, entropy, symmetry) for objective interpretability assessment—highlighting fractal dimension as the most robust discriminator of attention quality.
- **Win–Lose model forensics:** A new evaluation paradigm emphasizing pairwise analysis of correct and incorrect predictions, revealing complementary strengths rather than a single dominant model.

### General Limitations

The present study is subject to several limitations. The analytical approach, which relied on selected data subsets, may not be fully representative of the model’s general behavior. Future investigations would benefit from a comprehensive assessment of interpretability and performance across all training epochs and the complete dataset. The inherent imbalance in the dataset, particularly for rare lesion classes, constrains the statistical confidence of our findings; incorporating class-balancing methods is therefore a critical direction for future work. It is also important to note that the perceptual metrics (e.g., fractal dimension and symmetry) are predicated on specific image properties and may require recalibration for use with other imaging modalities.

### External Validation and Dataset Limitations

This study relies on publicly available datasets (e.g., DermaMNIST), which provide a standardized benchmark for reproducibility and comparison across architectures. However, the results may not fully capture the variability and complexity of real-world clinical data. Future work should validate the proposed framework on locally acquired hospital datasets, which would allow assessment of generalization to diverse patient populations, imaging conditions, and lesion presentations. Such clinical validation would strengthen confidence in deploying high-performing models in practical medical settings.

In summary, this study challenges the notion of a universal high-performing model and proposes an iterative explainable AI (XAI) framework for medical imaging—emphasizing architectural diversity, interpretability, and continual refinement as central to robust and transparent medical AI.

## Data Availability

This study uses publicly available datasets from the MedMNIST repository (https://medmnist.com/).

## Footnotes

1 https://pytorch.org/vision/stable/transforms.html

2 https://docs.scipy.org/doc/scipy/reference/generated/scipy.stats.entropy.html

3 https://en.wikipedia.org/wiki/Sobel_operator

4 https://www.statsmodels.org/dev/generated/statsmodels.stats.multitest.multipletests.html

## References

[1] Adebayo, J., Gilmer, J., Muelly, M., Goodfellow, I., Hardt, M., Kim, B.: Sanity checks for saliency maps. Advances in neural information processing systems **31** (2018). 10.48550/arXiv.1810.03292

[2] Ahmad, F., Zhang, X., Tang, Z., Sabah, F., Azam, M., Sarwar, R.: Deep deterministic policy gradients with a self-adaptive reward mechanism for image retrieval. The Journal of Supercomputing **81**(1), 336 (2025). 10.1007/s11227-024-06764-9

[3] Arun, N., Gaw, N., Singh, P., Chang, K., Aggarwal, M., Chen, B., Hoebel, K., Gupta, S., Patel, J., Gidwani, M., et al.: Assessing the trustworthiness of saliency maps for localizing abnormalities in medical imaging. Radiology: Artificial Intelligence **3**(6), e200267 (2021). 10.1148/ryai.2021200267 10.1148/ryai.2021200267 PMC8637231 34870212

[4] Bach, S., Binder, A., Montavon, G., Klauschen, F., Müller, K.R., Samek, W.: On pixel-wise explanations for non-linear classifier decisions by layer-wise relevance propagation. PloS one **10**(7), e0130140 (2015). 10.1371/journal.pone.0130140 26161953 10.1371/journal.pone.0130140 PMC4498753

[5] Backes, A.R., Casanova, D., Bruno, O.M.: A complex network-based approach for boundary shape analysis. Pattern Recognition **42**(1), 54–67 (2009). 10.1016/j.patcog.2008.07.006

[6] Benjamini, Y., Hochberg, Y.: Controlling the false discovery rate: a practical and powerful approach to multiple testing. Journal of the Royal statistical society: series B (Methodological) **57**(1), 289–300 (1995). 10.1111/j.2517-6161.1995.tb02031.x

[7] Block, K.T.: Subtle pitfalls in the search for faster medical imaging. Proceedings of the National Academy of Sciences **119**(17), e2203040119 (2022). 10.1073/pnas.2203040119 10.1073/pnas.2203040119 PMC9170040 35452309

[8] Chen, J.E., Chang, C., Greicius, M.D., Glover, G.H.: Introducing co-activation pattern metrics to quantify spontaneous brain network dynamics. Neuroimage **111**, 476–488 (2015). 10.1016/j.neuroimage.2015.01.057 25662866 10.1016/j.neuroimage.2015.01.057 PMC4386757

[9] Colliot, O., Thibeau-Sutre, E., Brianceau, C., Burgos, N.: Reproducibility in medical image computing: what is it and how is it assessed? Elsevier, Amsterdam (2025). 10.1016/B978-0-44-323761-4.00018-3

[10] Dzhezyan, G., Cecotti, H.: Symmetrical filters in convolutional neural networks. International Journal of Machine Learning and Cybernetics **12**(7), 2027–2039 (2021). 10.1007/s13042-021-01290-z

[11] Ferreira, G.B., Silva, P., Silva, R.: Elevating healthcare ai: Achieving efficiency and accuracy in medical applications with surrogate-based multiobjective compression of resnet50 cnns. In: Brazilian Conference on Intelligent Systems. pp. 137–151. Springer (2024). 10.1007/978-3-031-79038-6_10

[12] Gatys, L., Ecker, A.S., Bethge, M.: Texture synthesis using convolutional neural networks. Advances in neural information processing systems **28** (2015). 10.48550/arXiv.1505.07376

[13] Halder, A., Dalal, A., Gharami, S., Wozniak, M., Ijaz, M.F., Singh, P.K.: A fuzzy rank-based deep ensemble methodology for multi-class skin cancer classification. Scientific Reports **15**(1), 6268 (2025). 10.1038/s41598-025-90423-3 39979375 10.1038/s41598-025-90423-3 PMC11842842

[14] Hogeweg, L., Sánchez, C.I., Maduskar, P., Philipsen, R.H., van Ginneken, B.: Fast and effective quantification of symmetry in medical images for pathology detection: Application to chest radiography. Medical Physics **44**(6), 2242–2256 (2017). 10.1002/mp.12127 28134985 10.1002/mp.12127

[15] Johnson, A., Smith, B., Lee, C., Kim, D., Wang, E.: Hybrid neural networks with quantum transformations for enhanced image classification performance

[16] Kumar, S., Abdelhamid, A.A., Tarek, Z.: Visualizing the unseen: Exploring grad-cam for interpreting convolutional image classifiers. J. Full Length Artic **4**, 34–42 (2023). 10.54216/JAIM.040104

[17] Liu, H., Brailsford, T., Bull, L.: Exploring filter placement in convolutional layer topologies based on resnet for image classification. Machine Vision and Applications **36**(3), 54 (2025). 10.1007/s00138-025-01674-z

[18] Liu, H., Brailsford, T., Bull, L.: Resnet18 performance: Impact of network depth and image resolution on image classification. In: Proceedings of the 2024 8th International Conference on Advances in Artificial Intelligence. pp. 351–356 (2024). https://dl.acm.org/doi/10.1145/3704137.3704173

[19] Loy, G., Eklundh, J.O.: Detecting symmetry and symmetric constellations of features. In: Computer Vision–ECCV 2006: 9th European Conference on Computer Vision, Graz, Austria, May 7-13, 2006. Proceedings, Part II 9. pp. 508–521. Springer (2006). 10.1007/11744047_39

[20] Masters, B.R., Gonzalez, R.C., Woods, R.: Book Review: Digital Image Processing, Third Edition. Journal of Biomedical Optics **14**(2), 029901 (2009). 10.1117/1.3115362

[21] Nikolić, M., Janković, D., Stanimirović, A., Stoimenov, L.: The integration of explainable ai methods for the classification of medical image data. In: 2024 11th International Conference on Electrical, Electronic and Computing Engineering (IcETRAN). pp. 1–6. IEEE (2024). 10.1109/IcETRAN62308.2024.10645095

[22] Otsu, N., et al.: A threshold selection method from gray-level histograms. Automatica **11**(285-296), 23–27 (1975). 10.1109/TSMC.1979.4310076

[23] Peleg, S., Naor, J., Hartley, R., Avnir, D.: Multiple resolution texture analysis and classification. IEEE transactions on pattern analysis and machine intelligence (4), 518–523 (1984). 10.1109/TPAMI.1984.4767557 10.1109/tpami.1984.4767557 21869220

[24] Qi, Z., Khorram, S., Li, F.: Visualizing deep networks by optimizing with integrated gradients. In: AAAI. vol. 34, pp. 11890–11898 (2020). 10.1609/aaai.v34i07.6863

[25] Raghavan, K., Balasubramanian, S., Veezhinathan, K.: Explainable artificial intelligence for medical imaging: Review and experiments with infrared breast images. Computational Intelligence **40**(3), e12660 (2024). 10.1111/coin.12660

[26] Renard, F., Guedria, S., Palma, N.D., Vuillerme, N.: Variability and reproducibility in deep learning for medical image segmentation. Scientific Reports **10**(1), 13724 (2020). 10.1038/s41598-020-69920-0 32792540 10.1038/s41598-020-69920-0 PMC7426407

[27] Russ, J.C.: The image processing handbook (2006). 10.1201/9780203881095

[28] Selvaraju, R.R., Cogswell, M., Das, A., Vedantam, R., Parikh, D., Batra, D.: Grad-cam: Visual explanations from deep networks via gradient-based localization. In: Proceedings of the IEEE international conference on computer vision. pp. 618–626 (2017). ttps://doi.org/10.1007/s11263-019-01228-7

[29] Sendjasni, A., Traparic, D., Larabi, M.C.: Investigating normalization methods for cnn-based image quality assessment. In: 2022 IEEE International Conference on Image Processing (ICIP). pp. 4113–4117. IEEE (2022). 10.1109/ICIP46576.2022.9897268

[30] Shannon, C.E.: A mathematical theory of communication. The Bell system technical journal **27**(3), 379–423 (1948). 10.1002/j.1538-7305.1948.tb01338.x

[31] Taylor, R.P., Micolich, A.P., Jonas, D.: Fractal analysis of pollock’s drip paintings. Nature **399**(6735), 422–423 (2011). 10.1038/20833

[32] Yang, J., Subbotin, A., Zhukova, N.: Application of secure visual language model-based framework for collaborative analysis of dermatological images. In: 2025 XXVIII International Conference on Soft Computing and Measurements (SCM). pp. 443–447. IEEE (2025). 10.1109/SCM66446.2025.11060099

[33] Yang, J., Shi, R., Wei, D., Liu, Z., Zhao, L., Ke, B., Pfister, H., Ni, B.: Medmnist v2-a large-scale lightweight benchmark for 2d and 3d biomedical image classification. Scientific Data **10**(1), 41 (2023). 10.1038/s41597-022-01721-8 36658144 10.1038/s41597-022-01721-8 PMC9852451

[34] Zhou, X., Liu, W., Zhang, L., Zhang, X.: Boundary-sensitive adaptive decoupled knowledge distillation for acne grading: X. zhou et al. Applied Intelligence **55**(6), 426 (2025). 10.1007/s10489-025-06260-4

[35] Zhou, Y., Xu, M., Hu, Y., Lin, H., Jacob, J., Keane, P.A., Alexander, D.C.: Learning to address intra-segment misclassification in retinal imaging. In: Medical Image Computing and Computer Assisted Intervention–MICCAI 2021: 24th International Conference, Strasbourg, France, September 27–October 1, 2021, Proceedings, Part I 24. pp. 482–492. Springer (2021). 10.1007/978-3-030-87193-2_46

